# Platelet glycoprotein VI modulates leukocyte characteristics in sepsis‐induced systemic inflammatory response in male mice

**DOI:** 10.14814/phy2.70971

**Published:** 2026-06-16

**Authors:** Adam Corken, Jerry Ware, Keshari M. Thakali

**Affiliations:** ^1^ Department of Pediatrics University of Arkansas for Medical Sciences Little Rock Arkansas USA; ^2^ Arkansas Children's Research Institute Little Rock Arkansas USA; ^3^ Department of Physiology and Cell Biology University of Arkansas for Medical Sciences Little Rock Arkansas USA

**Keywords:** GPVI, inflammation, leukocytes, platelets, sepsis

## Abstract

Platelets are responsible for maintaining vascular integrity through the process of hemostasis with glycoprotein VI (GPVI) acting as a major receptor for platelet activation. Interestingly, platelet participation in inflammation is an evolving concept with few studies exploring GPVI in the framework of inflammation and even less in the context of the most severe inflammatory condition: sepsis. Here we utilized a GPVI deficient mouse model (GPVI^−/−^) to elucidate the role of GPVI in the formation of circulating platelet‐leukocyte aggregates (PLAs) and how GPVI driven modifications impact leukocyte phenotype and function under septic systemic inflammatory conditions. Using the cecal ligation and puncture (CLP) technique to model sepsis in male mice, we report GPVI‐mediated modification of several inflammatory characteristics. GPVI deficiency reduced platelet adhesion to neutrophils and monocytes during sepsis, resulting in reduced neutrophil and increased monocyte activation. Furthermore, monocytes exhibited altered polarization with GPVI deletion, raising the levels of circulating non‐classical monocytes. The loss of GPVI also elicited a significant reduction in the levels of circulating TNFα. Finally, sepsis‐induced mortality was significantly exacerbated as survival was dramatically reduced in GPVI^−/−^ mice over a five‐day period. These findings suggest that platelet GPVI impacts the inflammatory response during sepsis in a previously undocumented manner.

## 1 INTRODUCTION

Platelets are small, highly granulated cells that maintain the integrity of the circulatory network by surveying for vascular injury and rapidly aggregating at sites of damage to form a hemostatic plug (clot) (Jackson, 2007; van der Meijden & Heemskerk, 2019). This expedited response to vascular damage is the result of a platelet repertoire of surface receptors and granular cargo that permit accelerated adhesion, activation, and aggregation without the needed delay of de novo protein synthesis (Cimmino & Golino, 2013). While principally investigated for their clot forming ability that maintains vascular barriers (hemostasis) or occludes blood flow to major organs (thrombosis), platelets are functionally versatile with observed participation in various physiological pathways such as angiogenesis (Demirci Şahin et al., 2025), apoptosis (Repsold et al., 2017) tissue repair (Eisinger et al., 2018), adipogenesis (Liao et al., 2015) and tumorigenesis (Schaubaecher et al., 2024). Despite this diverse pathway involvement, outside of clot formation, platelets have routinely demonstrated preferential involvement in the immune/inflammatory process (Huilcaman et al., 2022; Rolfes et al., 2020). Indeed, platelets are frequently shown to either initiate, propagate, or modulate the immune response under a host of inflammatory conditions such that platelets are casually referenced as extensions of the immune system.

Though not canonically perceived as immune cells, platelets display a many functions pertaining to host defense and inflammation that parallels the traditional roles of leukocytes (Sonmez & Sonmez, 2017). Of note, platelets associate with microbes (Berthet et al., 2012; Portier & Campbell, 2021), directly engage in microbial destruction (Li et al., 2020; Yeaman, 2014), stimulate both innate (Mantovani & Garlanda, 2013) and adaptive immune cells (Sprague et al., 2008), facilitate leukocyte polarization (Saris et al., 2021; Uchiyama et al., 2021), and elicit specific immune functions such as Neutrophil Extracellular Trap (NET)osis (Caudrillier et al., 2012) and antigen presentation (Chapman et al., 2012). Because platelets are involved in a myriad of immune settings, the question arises as to their involvement in the most severe presentation of inflammation: sepsis. Sepsis initiates in a typical manner as an inflammatory response resulting from a (bloodborne) microbial infection (Jarczak et al., 2021). However, sepsis deviates from normal microbial clearance in its heightened, systemic state of inflammation that persists even in the absence of the initiating infection (Campanelli et al., 2022; Contou et al., 2016). A fully recognized ‘sepsis’ diagnosis is reached when these underlying conditions result in the dysfunction of various organ systems (Singer et al., 2016). Despite improvements in care, sepsis outcomes remain dire as 38%–46.5% of individuals who become septic will die as a result (Shankar‐Hari et al., 2016; Vincent et al., 2019). Moreover, it is estimated that one out of every three mortalities within hospitals are the direct result of sepsis (Rhee et al., 2017). The severity of sepsis and its impact on patient outcomes has driven an increased focus on uncovering the pertinent mechanisms underlying this disease's pathophysiology (Morrison et al., 2025).

The stagnation in sepsis mortality reduction has pivoted investigations away from the exogenous instigating microbe towards the host's endogenous immunological response. Accordingly, our lab previously demonstrated that the platelet‐specific receptor glycoprotein (GP)Ib‐IX‐V, principally known for its critical role in maintaining hemostasis, also modulates neutrophil and monocyte phenotype in a murine model of sepsis, which directly impacts the inflammatory response (Corken et al., 2014). This discovery led us to speculate whether other platelet receptors, such as GPVI, known primarily for hemostasis/thrombosis, exhibit functional duality as mediators of inflammation. GPVI principally serves as a node of platelet activation through the binding of its primary ligand collagen, resulting in degranulation and morphological changes that reinforce platelet aggregation and stable clot formation (Lockyer et al., 2006; Massberg et al., 2003). However, GPVI has also demonstrated immune functionality as it promotes the formation of pro‐inflammatory microparticles (Boilard et al., 2010). Notably, GPVI is also a counter receptor for the leukocyte transmembrane glycoprotein basigin (EMMPRIN, CD147), though the nature of the GPVI/basigin axis is unresolved (Muramatsu, 2016; Seizer et al., 2009). Here we investigated the contribution of GPVI to sepsis‐induced systemic inflammation. Utilizing a GPVI deficient mouse model, we recapitulated sepsis via cecal ligation and puncture (CLP) procedure to elucidate changes to the inflammatory profile stemming from GPVI loss (Dejager et al., 2011; Li et al., 2018). Of note, recent insights outline sexual dimorphism in sepsis morbidity and mortality, with males exhibiting significantly greater vulnerability to sepsis burden (Hu et al., 2025; Lakbar et al., 2023). Bearing this in mind, we chose to study males exclusively for our initial exploration into the GPVI contribution to systemic inflammation in the population most vulnerable to sepsis morbidity and mortality. We provide the results of our study highlighting GPVI‐dependent alterations in leukocyte characteristics and survival outcomes in a model of sepsis in male mice.

## 2 MATERIALS AND METHODS

### 2.1 Animals and experimental design

All animal procedures were directed under the conditions approved by the UAMS Institutional Animal Care and Use Committee. The generation and characterization of the mouse GPVI^−/−^ strain has been described in detail (Kato et al., 2003). Briefly, the disruption of GPVI expression was achieved by the insertion of a premature stop codon (TGA) followed by a XhoI restriction sequence and a neo^r^ cassette in the immediate 3′ position to the start codon (ATG) located within exon I. The absence of GPVI expression does not affect the expression of other platelet surface receptors such as GPIb‐IX‐V or GPIIb/IIIa nor does it alter circulating platelet counts or morphology. The GPVI^−/−^ strain has been thoroughly backcrossed (>10 generations) onto the C57Bl/6J background to represent a congenic strain. Wild‐type (C57Bl/6J) (WT) male and female mice were purchased from Jackson Laboratories to establish breeding harems. All harems consisted of two females and one male to generate the necessary offspring for experimentation. Breeding harems were fed Select Mouse 50 IF/95 breeder chow (PicoLab: 3005973‐220). Breeding harems and offspring were housed in opaque, polypropylene cages that rested on stainless steel racks in ventilated, climate‐controlled storage rooms within the University of Arkansas for Medical Sciences vivarium located within the Biomedical Research Building I. Male offspring were weaned at 4 weeks of age and housed together in similar cage conditions to the breeding harem. Offspring were fed Select Rodent 50 IF/6F chow (PicoLab: 3005954‐220). Post‐weaning cage conditions did not exceed 5 mice per group. Mice 22–24 weeks‐of‐age were used for the following experiments described below. For all procedures involving euthanasia, euthanasia was conducted via overdosing inhalation of CO_2_ followed by cervical cord dislocation.

### 2.2 Cecal ligation and puncture (CLP)

CLP surgery was performed as previously described (Toscano et al., 2011). Briefly, mice were deeply anesthetized using 1%–3% isoflurane in a vaporization chamber. Once immobilized, animals were transferred to a warming pad, and the continued administration of isoflurane was conducted using a nose cone extension of the vaporizer. The abdomen was shaved and sterilized using 70% ethanol before a midline incision was made first the epidermis and then in the abdominal wall thereby exposing the peritoneal content. The cecum was then removed from the peritoneal cavity and approximately 1 cm was ligated using 4–0 silk sutures (Ethicon: 683G). A 22‐gauge needle was used to puncture the cecum through and through, followed by gentle massaging allowing for a small amount of cecal material to extrude from the puncture sites. The cecum was then returned to the peritoneal cavity and anchored to the abdominal wall via suturing. The incision in the abdominal wall was closed first followed by the epidermis using the same sutures for the initial cecal ligation. 1 mL of 37°C isotonic saline was administered intraperitoneally before the mouse was revived. For sepsis survival experiments, mice were continually monitored for a 5‐day period to assess septic mortality. All remaining animals were euthanized after a 5‐day period. For cytometry, hemodynamic and serological assays conducted 24 h following the induction of sepsis, animals were euthanized following the harvesting of the desired tissue sample.

### 2.3 Whole blood collection and cell counting

Mice were anesthetized using 1%–3% isoflurane in 100% O_2_ administered via vaporization chamber (E‐Z Systems EZ‐150C Classic Anesthesia Machine). Whole blood was collected by the insertion of a heparinized capillary tube into the retro‐orbital sinus (Fisher Scientific, Fisherbrand Color‐Coded Capillary Tubes: 22‐260‐950). Blood was collected in 1.5 mL centrifuge tubes containing a 3.8% sodium citrate solution (Fisher Scientific, Sodium Citrate Dihydrate: BP327‐500). Blood samples were kept individually at room temperature and never pooled. Blood cell counts were performed using a scil Vet abc Plus+ instrument (Scil Animal Care Company).

### 2.4 Evans blue blood vessel permeability

Vascular permeability was assessed using Evans Blue dye (Sigma Aldrich: E2129‐10G) as documented previously (Radu & Chernoff, 2013). Evans Blue dye was dissolved in sterile PBS (Sigma Aldrich: P14417‐50TAB) to yield a 0.5% solution. 200 μL Evans Blue dye was administered via retro‐orbital injection into anesthetized mice. 15 min was allowed to pass for optimal dye dissemination throughout the vasculature network before animals were euthanized. A section of the liver, both lungs, and both kidneys were removed and weighed before being placed in 500 μL of formamide (Fisher Scientific: BP228‐100). Organs were then incubated at 55°C for 24 h to allow for Evans Blue diffusion into formamide. The absorbance of Evans Blue in formamide was recorded using a microplate spectrophotometer (BMG Labtech, POLARstar Omega microplate reader) and quantified by referencing a standard curve. The quantity of Evans Blue was then normalized to the mass of the individual organ.

### 2.5 Immunological reagents

The following antibodies were utilized for flow cytometry and imaging cytometry assays. BV786 conjugated anti‐mouse CD41 (740903) and BV605 conjugated anti‐mouse CD62P [P‐Selectin] (740358) antibodies were purchased from BD Biosciences. FITC conjugated anti‐mouse Gr‐1 (11‐5931‐82), PE conjugated anti‐mouse CD115 (12‐1152‐82), and Alexa Fluor 700 conjugated anti‐mouse CD11b (56‐0112‐82) were purchased from Thermo Fisher Scientific.

### 2.6 Flow cytometry analysis

A 25 μL aliquot of whole blood was transferred to a 5 mL polystyrene round bottom tube. The sample was diluted with 75 μL of PBS and 25 μL of flow cytometry antibody master mix was then added to the sample followed by gentle shaking at room temperature for 30 min. The antibody master mix consisted of 1.0 μL anti‐CD41, 1.0 μL anti‐CD62P, 0.5 μL anti‐CD115, 0.25 μL anti‐CD11b and 0.25 μL anti‐Gr‐1 and was diluted to a final volume of 25 μL with PBS. After 30 mins 400 μL of BD FACS Lysing solution (Waters Bioscience #: 349202) was added to each sample to fix and lyse the red blood cell population. Samples were shaken for an additional 15 min at room temperature. Sample data was then collected using a BD LSRFortessa with acquisition parameters set to collect 25,000 leukocyte events based on forward and side scattering. Sample data was analyzed using FlowJo v10 software as previously described (Corken et al., 2014).

Leukocyte subcategorization was conducted by establishing a cell gate based on size (FSC) and granularity (SSC) characteristics to exclude instrument noise and events outside the size range of typical blood cells (Figure S1). Next, CD115 and Gr‐1 were displayed on the dot plot axes and events that fluoresced positively for CD115 were denoted as monocytes (CD115^+^) while events that were CD115 negative but Gr‐1 positive were considered neutrophils (Gr‐1^+^/CD115^−^). While Gr‐1 is a general granulocyte marker, we characterized these granulocyte events as neutrophils because neutrophils comprise the vast majority of the granulocyte population. After the neutrophil and monocyte populations were established, the presence of platelets was determined using CD41 fluorescence. By exclusionary gating from all other events, platelet‐bound neutrophils and monocytes were referenced to the total neutrophil and monocyte populations to determine the percentage of CD41^+^ events for each leukocyte subpopulation.

Mac‐1 (CD11b/CD18) expression was determined for both the neutrophil and monocyte population by assessing the geometric mean fluorescent intensity (MFI) of CD11b within each gate. Average CD11b fluorescence was not only generated for the neutrophil/monocyte population as a whole but also for CD41^+^ and CD41^−^ subgroups. These values were used to generate a ratio of CD11b expression for CD41^+^ over CD41^−^ events which underwent a log2 transformation to better illustrate the direction of CD11b surface expression induced by platelet binding. Furthermore, monocyte expression of Gr‐1 was used in the determination of the sub‐categories of classical and non‐classical monocytes. Classical monocytes were noted as CD115^+^ events which were Gr‐1^High^ while non‐classical monocytes were CD115^+^ and Gr‐1^Low^.

Platelet P‐selectin expression analysis occurred subsequently by returning to the total (ungated) population of events. CD41 fluorescence and forward scattering parameters were displayed and CD41^+^ events were gated with the remainder of events excluded from further analysis (Figure S2). Focusing on platelet positive events, the parameter axes were changed to CD115 and Gr‐1 fluorescence. Gates were drawn around Gr‐1^+^/CD115^−^, CD115^+^, and Gr‐1^−^/CD115^−^ populations. The Gr‐1^+^/CD115^−^ population was interpreted as platelet–neutrophil aggregates, while CD115^+^ events noted as platelet‐monocyte aggregates and Gr‐1^−^/CD115^−^ events taken as unassociated platelets. The geometric MFI for P‐selectin within each group was then recorded. Like the leukocyte analysis, P‐selectin values were also expressed as log2 transformed ratios of neutrophil/monocyte bound platelets relative to unbound platelets to highlight the alteration in P‐selectin expression on platelets resulting from aggregation with leukocytes.

### 2.7 Imaging flow cytometry

For imaging cytometry, a 25 μL aliquot of whole blood was stained in as previously described with a few modifications. First, the antibody master mix for imaging cytometry omitted P‐selectin and CD11b antibodies as the imaging cytometer had significantly fewer channels for detecting fluorescent signals relative to a flow cytometer. Second, after staining and fixation, samples were centrifuged at 1000 rcf × g for 5 min, the supernatant discarded and the pellet reconstituted in 50 μL of PBS. Sample data was recorded on an Amnis ImageStream^X^ Mark II Imaging Flow Cytometer. The acquisition threshold was set to collect 1000 neutrophil (Gr‐1^+^/CD115^−^) and monocyte (CD115^−^) events. Sample data was analyzed using Amnis IDEAS software wherein a gating strategy similar to that of our flow cytometry analysis. Briefly, Gr‐1 and CD115 staining was used to establish the neutrophil and monocyte populations. Next, CD41^+^ staining was used to determine the platelet‐leukocyte aggregate population. For each platelet‐positive neutrophil and monocyte event, the accompanying microscopic image was manually analyzed, and the number of adherent platelets was recorded. After tallying bound platelet values for all CD41^+^ neutrophil and monocyte events within a single sample, the measurements were averaged to yield a single value for each leukocyte subpopulation for each sample.

### 2.8 TNFα serological analysis

Whole blood collection for serological analysis was collected as previously described but with the following modifications. First, un‐heparinized capillary tubes were used to facilitate the blood draw from the retro‐orbital sinus. Additionally, blood was collected in 1.5 mL centrifuge tubes that were absent of any anti‐coagulant solution. Blood was kept at room temperature for 2 h to allow for clotting and subsequently centrifuged at 2000 rcf × g for 20 min to pellet the clot. The serum supernatant was transferred to an alternate 1.5 mL tube and then snap frozen in liquid nitrogen. Samples were then stored at −80°C until further analysis. Once all the required samples had been collected, serum TNFα concentration was measured using a commercially available ELISA kit (Thermo Fisher Mouse TNF alpha ELISA Kit: BMS607‐3).

### 2.9 Statistical analysis

A Mann–Whitney *U* test was utilized to compare each variable between WT and GPVI^−/−^ groups for all statistical analysis except for CLP survival. For CLP survival specifically, a Mantel‐Cox (log‐rank) test was used to compare survival outcomes between WT and GPVI^−/−^ cohorts. In all cases, a *p*(value) less than or equal to 0.05 was considered to be statistically significant. Previous statistical comparisons of circulating platelet‐leukocyte aggregate numbers and CD11b expression informed the power analysis for this study. Based on an expected between‐group difference of CD11b expression of 4000 geometric MFI and a within‐group standard deviation of 2000 geometric MFI, a minimum sample size of 18 mice per group was calculated to provide a power of 80% using a two‐tailed statistical analysis controlling for type I error of 0.05 (December 2024, Piface v1.76).

## 3 RESULTS

### 3.1 GPVI deletion alters septic survival in male mice

**FIGURE 1:**
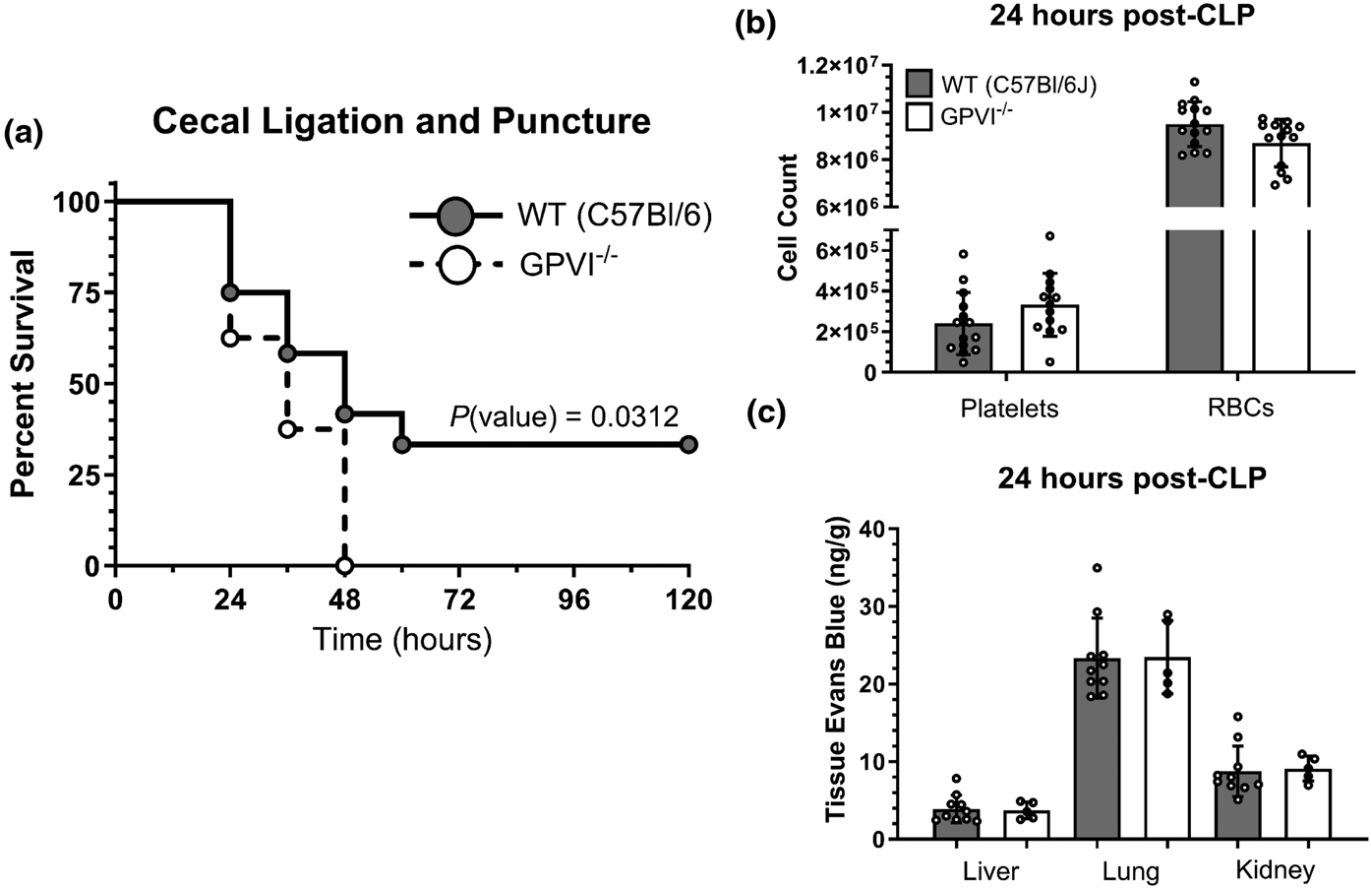
CLP induced sepsis survival and hemodynamic parameters. (a) Sepsis mortality was observed over a 5‐day period to determine GPVI‐related contribution to disease burden. *N* = 12 (WT); 16 (GPVI^−/−^). (b) Cell counts for circulating platelets and RBCs 24 h post‐CLP induction. *N* = 14 (WT); 13 (GPVI^−/−^). (c) Quantitation of Evans blue dye within the liver, lungs and kidneys 24 h after conducting CLP and following intravenous administration of Evans blue. *N* = 10 (WT); 5 (GPVI^−/−^).

We first compared sepsis survival outcomes of GPVI deficient (GPVI^−/−^) mice to WT controls. Following CLP, mice were continuously monitored over a 5‐day period (120 h) to approximate time of mortality to the nearest 12‐h time interval (Figure 1a). We observed a significant increase in disease burden in the GPVI^−/−^ cohort as lack of GPVI was associated with accelerated sepsis‐induced mortality compared to WT. All GPVI^−/−^ mice died as a result of sepsis within 48 h, while approximately one third of WT survived to the study's conclusion (5 days). As sepsis pathophysiology is the direct result of not only inflammatory but hemostatic dysregulation, we next assessed hemodynamic perturbations (Levi & van der Poll, 2017). We evaluated the levels of both platelets and red blood cells (RBCs) in circulation in a separate cohort from the mortality study 24 h after CLP (Figure 1b). Surprisingly, we did not observe any difference in either platelet or RBC count when comparing GPVI^−/−^ to WT samples. We next quantified Evans Blue dye content in several major organs, following intravenous administration of the dye 24 h after CLP (Figure 1c). When comparing Evans Blue content in liver, lung or kidneys from septic animals, GPVI^−/−^ did not differ from WT, suggesting that the difference in survival was not due to GPVI‐related exacerbation of vascular permeability in sepsis.

### 3.2 Baseline assessment of GPVI contribution to platelet‐leukocyte aggregates

**FIGURE 2:**
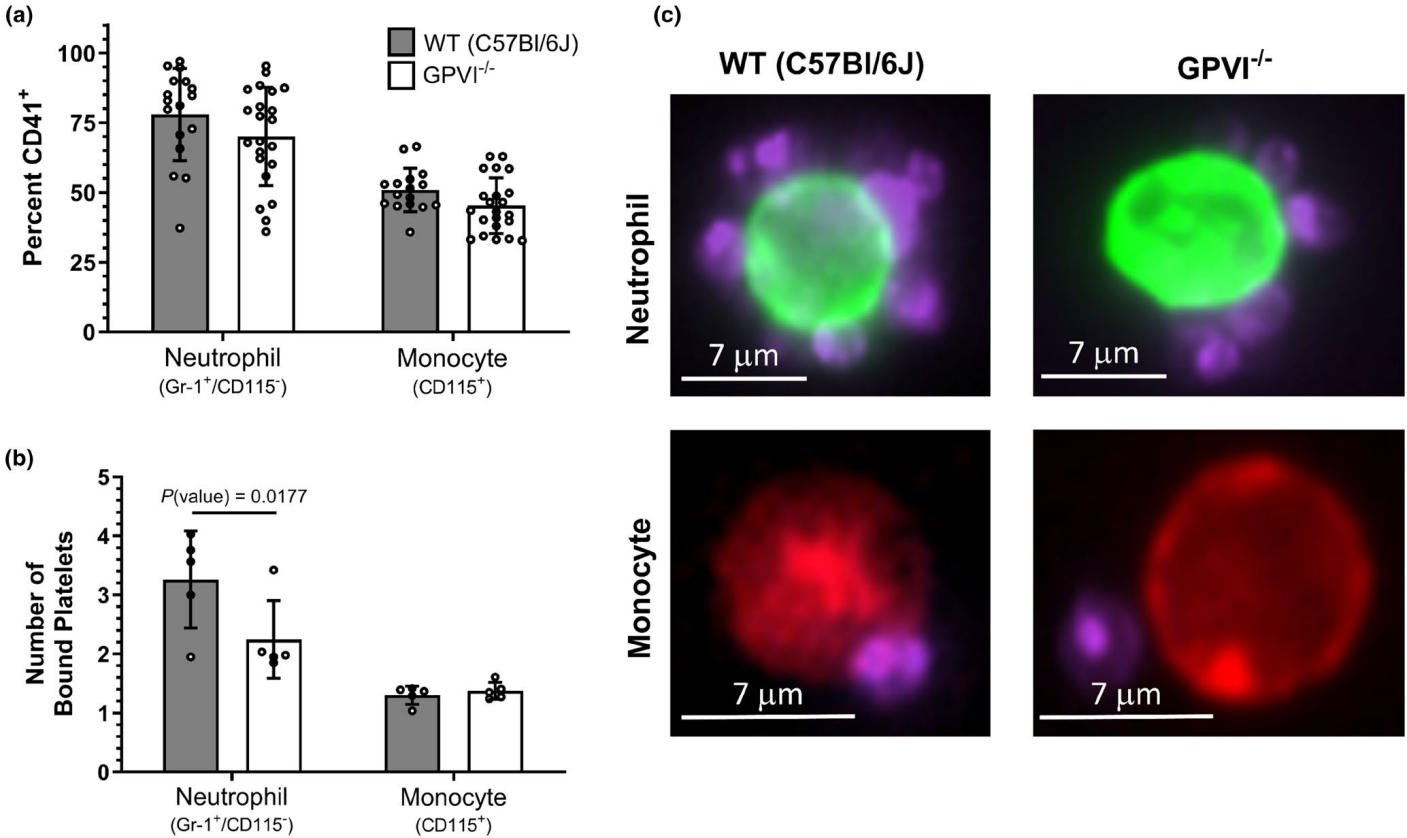
Traditional flow cytometry and imaging cytometry characterization of circulating platelet‐leukocyte aggregate characteristics at baseline. (a) The percentage of platelet bound (CD41^+^) neutrophils and monocytes within the WT and GPVI^−/−^ cohorts relative to the entire leukocyte subpopulation. *N* = 18 (WT); 25 (GPVI). (b) The average number of platelets bound to individual, circulating neutrophils and monocytes from WT and GPVI whole blood samples. *N* = 5 (WT and GPVI). (c) A representative imaging cytometer image illustrating the association of platelets (purple) to neutrophils (green) and monocyte (red) in circulation from both WT and GPVI^−/−^ samples.

Since there were no differences in hematologic components or vascular permeability as a result of GPVI deletion, we pursued potential alterations in the inflammatory pathway stemming from GPVI loss. As platelets associate with leukocytes in circulation and this resultant aggregation influences leukocyte functionality, we began our exploration of the GPVI/inflammatory axis by documenting PLA levels in both WT and GPVI^−/−^ mice (Cerletti et al., 2012; Ghasemzadeh & Hosseini, 2013; Han et al., 2025; Lam et al., 2015). Under normal conditions, GPVI deletion does not influence the percentage of circulating neutrophils or monocytes bound by platelets as determined by flow cytometry (Figure 2a). Though traditional flow cytometry is crucial as a first step to determine the magnitude of leukocytes associated with platelets, it merely indicates the subset within a population that is CD41^+^, meaning at least one platelet is present. As this did not address the number of platelets bound to leukocytes, we next utilized imaging cytometry to elaborate on our previous findings by characterizing the number of platelets adherent to each leukocyte sub‐category. Interestingly, we found that while GPVI deletion does not impact the total number of neutrophils bound by platelets, it does diminish the number of platelets bound to each neutrophil (Figure 2b). This same pattern was not displayed by monocytes in the absence of GPVI as monocytes tethered to only one platelet on average, regardless of the presence/absence of GPVI. Representative images of platelet–neutrophil and platelet‐monocyte aggregates for both WT and GPVI^−/−^ samples at baseline are outlined in Figure 2c.

### 3.3 Leukocyte and platelet activation status resulting from GPVI deletion at baseline

**FIGURE 3:**
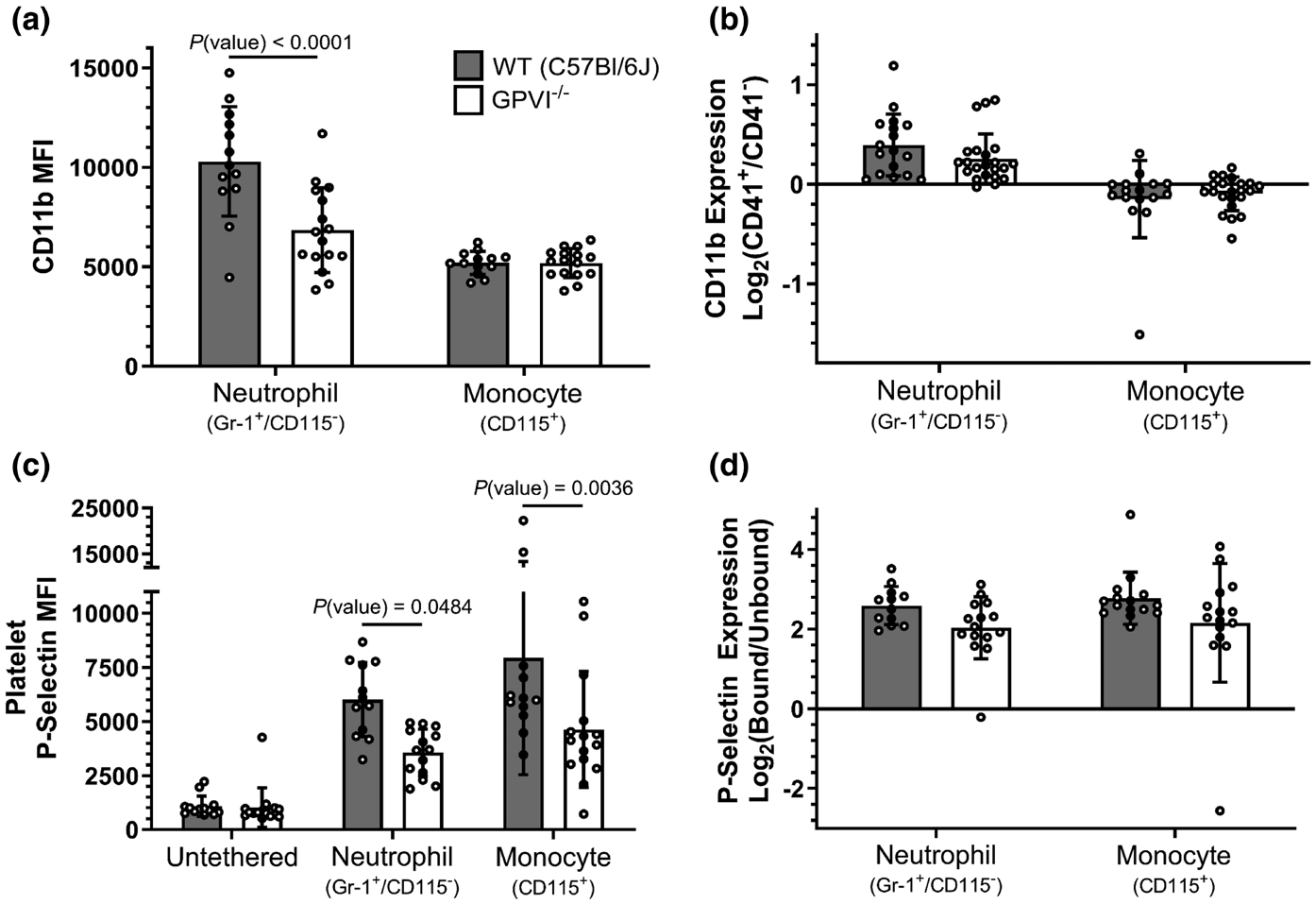
Activation characteristics of circulating leukocytes and platelets. (a) Surface expression profiles of CD11b for the total neutrophil and monocyte population and (b) the ratio of CD11b expression for platelet‐bound (CD41^+^) to platelet‐free (CD41^−^) neutrophils and monocytes. (c) P‐selectin surface expression on free (untethered) platelets and platelets associated with neutrophils and monocytes in circulation. (d) The ratio of P‐selectin expression of platelets bound to neutrophils and monocytes in relation to free platelets. *N* = 18 (WT); 25 (GPVI^−/−^).

To discern if GPVI might influence leukocyte or platelet activation metrics resulting from aggregation, we measured leukocyte activation by quantifying the surface expression (fluorescence) levels of CD11b. CD11b is the α‐subunit of the integrin Mac‐1 which is expressed on a variety of immune cells, including neutrophils and monocytes (Morgan et al., 2019). We noted a significant decrease in CD11b expression on GPVI^−/−^ neutrophils indicating that GPVI plays some part in neutrophil stimulation under baseline conditions (Figure 3a). However, this same phenomenon was not observed in the monocyte population. Next, we compared the relative shift in CD11b expression on neutrophils and monocytes caused by platelet binding by normalizing expression to the values of unassociated counterparts. We observed that platelet binding to neutrophils facilitated an increase in CD11b expression and that the degree of CD11b expressional change did not differ between WT and GPVI^−/−^ samples (Figure 3b). Thus, it appears that on average, platelet binding proportionally increases neutrophil activation independent of GPVI expression but overall activation within the neutrophil population is diminished in the absence of GPVI. For monocytes, platelet binding had minimal effect on CD11b levels relative to unbound monocytes.

Additionally, we examined the effect of aggregate formation on platelet activation status and how GPVI might influence this metric by quantifying platelet surface expression (i.e., fluorescence) of P‐selectin on neutrophil and monocyte bound platelets as well as non‐associated platelets (Figure 3c). CD41^+^ events that were negative for both Gr‐1 and CD115 were referred to as free or untethered platelets. GPVI deletion resulted in a significant reduction in activation, as indicated by P‐selectin expression, for platelets associated with neutrophils and monocytes in circulation. For unassociated platelets, the loss of GPVI had no impact on P‐selectin expression. We likewise explored the relative change in platelet activation expression by normalizing P‐selectin expression on leukocyte‐bound platelets to free platelets (Figure 3d). Platelet associations with neutrophils and monocytes caused an increase in activation relative to unaffiliated platelets. However, WT and GPVI^−/−^ samples were indistinguishable, indicating that GPVI does not play a role in modulating platelet activation as a result of leukocyte binding under normal conditions. Furthermore, the degree of activation was similar regardless of whether the platelet was bound to a neutrophil or a monocyte. Nonetheless, despite GPVI having no influence on the proportional increase in activation for leukocyte‐associated platelets relative to free platelets, it is responsible for significantly increased levels of total activation for platelets bound to neutrophils/monocytes.

### 3.4 GPVI impact on circulating PLAs following sepsis induction

**FIGURE 4:**
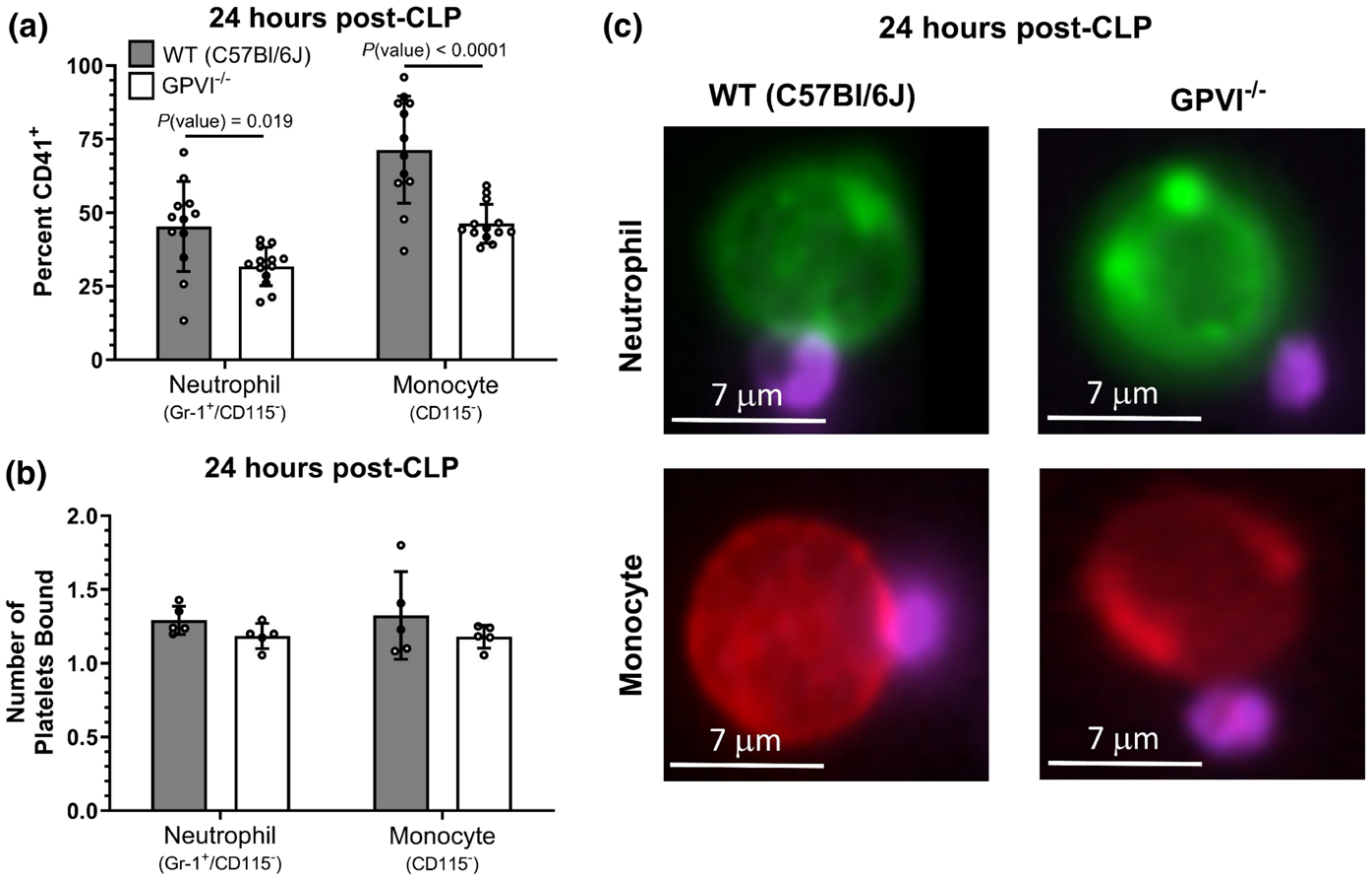
Analysis of circulating platelet‐leukocyte aggregate characteristics 24 h post‐CLP. (a) The average percentage of platelet‐associated (CD41^+^) neutrophils and monocytes relative to the total population under septic conditions for both WT and GPVI^−/−^ samples. *N* = 14 (WT); 13 (GPVI^−/−^). (b) The average number of platelets adhered to individual neutrophils and monocytes in septic blood samples form WT and GPVI^−/−^ cohorts. *N* = 5 (WT and GPVI^−/−^). (c) Representative images collected from an imaging cytometer depicting platelet association with neutrophils and monocytes in circulation following sepsis induction.

After determining baseline leukocyte and platelet characteristics stemming from GPVI deletion, we examined the impact of GPVI on these same parameters during a state of systemic inflammation following cecal ligation and puncture. While the absence of GPVI had no discernable impact on circulating PLA levels prior to sepsis, we observed significant differences in both platelet–neutrophil and platelet‐monocyte associations between WT and GPVI^−/−^ 24 h post‐CLP (Figure 4a). Indeed, the absence of GPVI resulted in a significant reduction in the percentage of both neutrophils and monocytes bound by platelets under septic conditions. Interestingly, when compared to non‐septic conditions, both WT and GPVI^−/−^ exhibited a reduction in the percentage of platelet–neutrophil associations after sepsis induction, albeit with WT displaying significantly higher levels relative to GPVI^−/−^. For the monocyte population, sepsis did not diminish the percentage associated with platelets. On the contrary, WT samples displayed a sizeable increase in the percentage of platelet‐monocyte associations relative to baseline conditions while GPVI^−/−^ samples appeared unchanged. This resulted in GPVI^−/−^ samples having significantly less circulating levels of platelet/monocyte associations relative to WT under septic conditions. When we assessed the number of adherent platelets per neutrophil/monocyte, we saw that sepsis did not alter the platelet‐monocyte ratio from baseline and that GPVI similarly had no impact (Figure 4b). However, for the neutrophil population, the onset of sepsis greatly altered the platelet–neutrophil ratio such that on average only one platelet was adherent per neutrophil when previously multiple platelets were bound under baseline conditions. WT and GPVI^−/−^ samples exhibited a comparable reduction in the platelet‐to‐neutrophil ratio under septic conditions insofar as the two groups were significantly different. Truly, the onset of sepsis mitigated any previous discrepancies in the ratio of adherent platelets to neutrophils resulting from GPVI deletion (Figure 4c). Despite this, GPVI did appear to significantly contribute to the binding of platelets to neutrophils and monocytes in total. Flow cytometry plots of representative samples outlining the method for determining levels of specific PLAs can be found in Figure S3.

### 3.5 GPVI's influence on platelet and leukocyte activation states during sepsis

**FIGURE 5:**
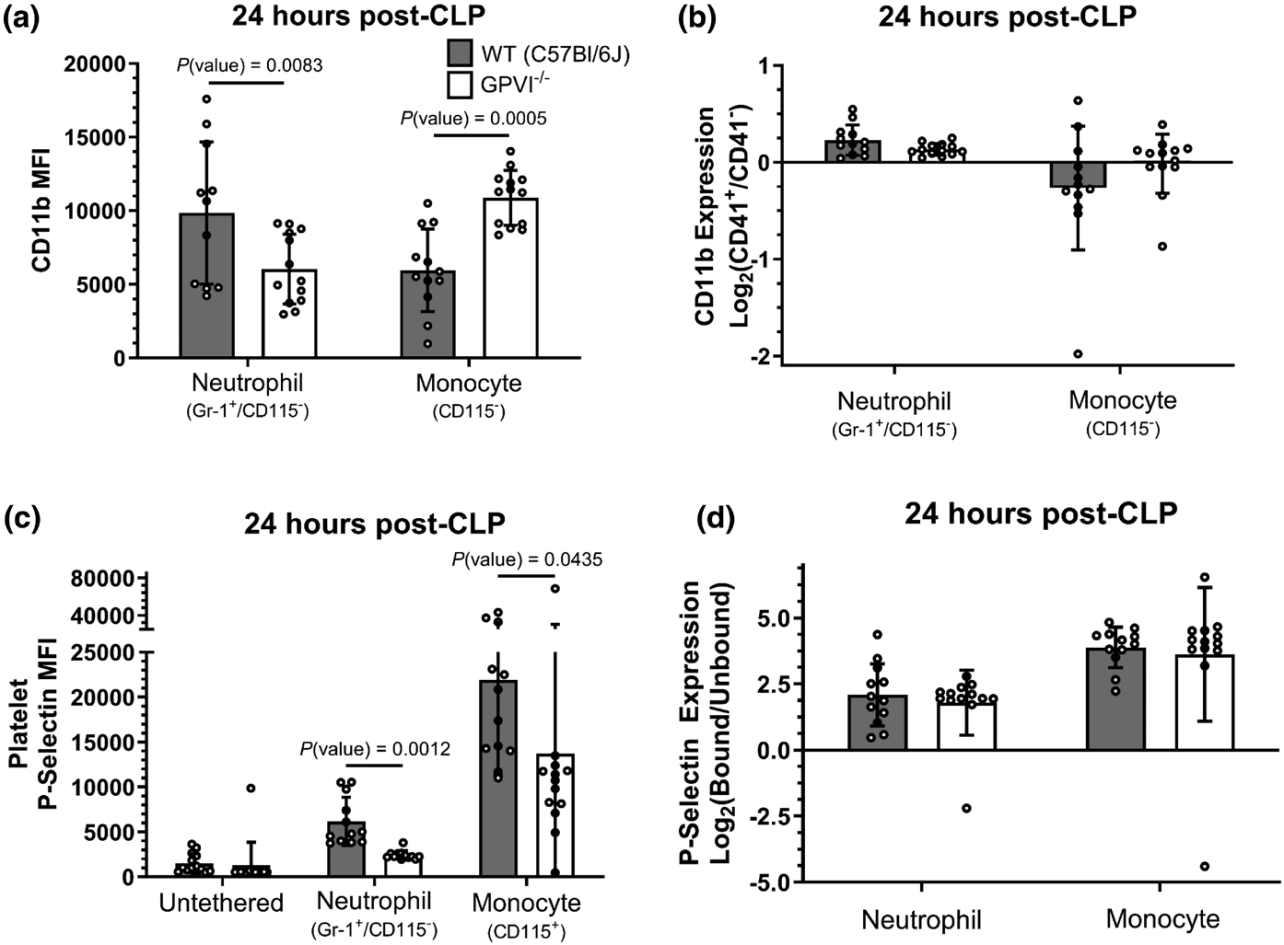
Leukocyte and platelet surface activation marker expression 24 h after sepsis induction. (a) Total neutrophil and monocyte surface CD11b expression. (b) The ratio of CD11b expression of platelet bound (CD41^+^) relative to platelet free (CD41^−^) neutrophils and monocytes. (c) P‐selectin expression for unbound platelets and for those aggregated to neutrophil and monocytes. (d) The ratio of P‐selectin expression on neutrophil and monocyte bound platelets in relation to unbound platelet P‐selectin expression. *N* = 14 (WT); 13 (GPVI^−/−^).

After elucidating the impact of GPVI deletion on septic PLA characteristics, we then evaluated the impact of GPVI loss on both leukocyte and platelet activation status during sepsis. Again, utilizing CD11b as a marker of leukocyte activation, we saw that GPVI^−/−^ neutrophils displayed significantly diminished levels of activation relative to WT (Figure 5a). Of note, these levels for both groups were comparable to those observed in baseline conditions. For monocytes, GPVI deletion elicited a significant increase in overall monocyte activation. Referencing these values to baseline conditions, we saw that sepsis did not evoke changes in WT monocyte CD11b expression but did so in GPVI^−/−^ derived monocytes. When assessing the relative change in CD11b expression facilitated by platelet adherence, we observed that platelet binding slightly increased neutrophil activation while having a negligible impact on monocyte activation during sepsis (Figure 5b). Moreover, WT and GPVI^−/−^ were indistinguishable for these metrics. While platelet adhesion caused similar proportional changes in CD11b expression for neutrophils and monocytes independent of GPVI, GPVI did impact total, overall CD11b expression by increasing neutrophil and minimizing monocyte expression in septic mice.

Using by P‐selectin to visualize platelet activation under septic conditions revealed diminished levels of activation for platelets bound to both neutrophils and monocytes in the absence of GPVI (Figure 5c). Untethered and neutrophil‐bound platelets did not differ in CD11b expression when referencing septic samples to baseline, regardless of GPVI status. However, both septic WT and GPVI^−/−^ monocyte‐bound platelets have dramatically increased CD11b levels relative to baseline. Assessing the relative direction of P‐selectin expression change elicited by platelet coupling to leukocytes demonstrated that platelet adhesion to both neutrophils and monocytes enhanced activation (Figure 5d). This pattern of enhancement during sepsis did not appear to differ in degree from our observations under baseline conditions. Representative figures for both leukocyte CD11b and platelet P‐selectin expression following sepsis induction are located in Figure S4.

### 3.6 GPVI driven modulation of monocyte polarity under septic conditions

**FIGURE 6:**
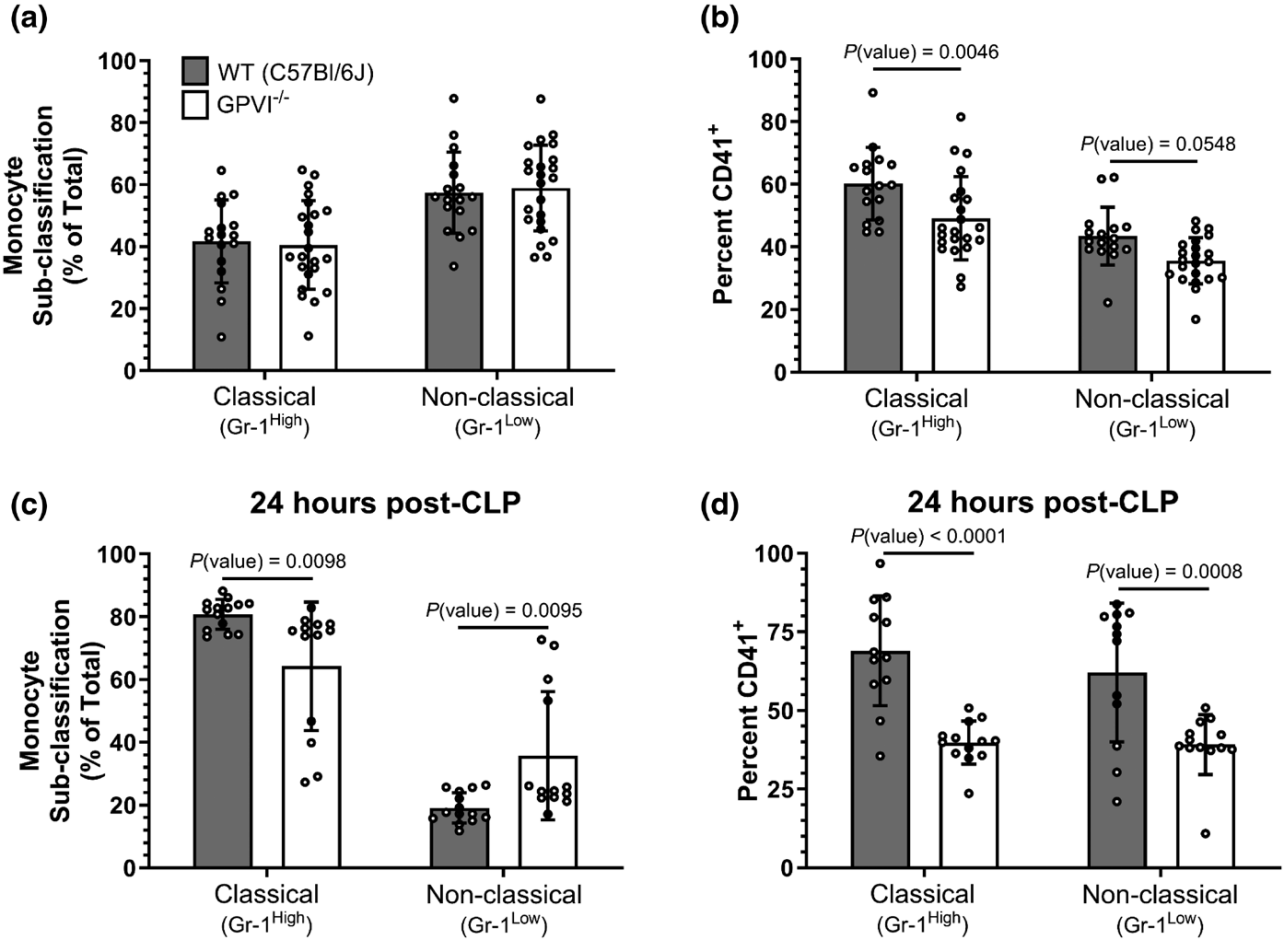
Monocyte sub‐classification and platelet association prior to and 24 h following sepsis initiation. (a) The baseline distribution prior to CLP of classical and non‐classical monocyte sub‐types relative to the total monocyte population. (b) The percentage of classical and non‐classical monocytes associated with platelets at before sepsis induction. (c) The proportion of classical and non‐classical monocytes relative to the total monocyte population 24 h after CLP. (d) The percentage of platelet‐associated classical and non‐classical monocytes 24 h post CLP. Baseline conditions: *N* = 18 (WT); 25 (GPVI). Septic conditions: *N* = 14 (WT); 13 (GPVI^−/−^).

As monocytes are a heterogeneous population, we next evaluated the proportion of the classical and non‐classical subcategories of monocytes influenced by GPVI deletion. Sub‐categorization was based on the expression of the surface marker Ly6C which is the antigen target of the Gr‐1 antibody (Dunay et al., 2008). Monocytes expressing positive (+) or high Ly6C (Gr‐1) levels represent the classical sub‐category while Ly6C (Gr‐1) negative (−) or low cells constitute non‐classical monocytes (Trzebanski et al., 2023). At baseline, classical (Gr‐1^High^) and non‐classical (Gr‐1^Low^) monocyte distributions did not differ when comparing WT to GPVI^−/−^ samples, with the non‐classical monocyte being the most prevalent type (Figure 6a). Following sepsis induction, there was a dramatic uptick in classical monocyte population such that it was the overwhelming majority for both WT and GPVI^−/−^ (Figure 6b). Interestingly, only after sepsis induction did WT and GPVI^−/−^ diverge in monocyte content with GPVI deletion resulting in significantly less classical and inversely increased non‐classical monocyte relative to WT. Of note, the percentage of classical monocytes associated with platelets was significantly reduced in the absence of GPVI at baseline (Figure 6c). The association of non‐classical monocytes to platelets trended to be diminished in GPVI^−/−^ samples although it failed to reach statistical significance. While our previous observation of the broad monocyte population at baseline indicated GPVI expression had no impact on platelet associations, it seems apparent that GPVI affected platelet aggregate formation with specific monocyte sub‐categories. Following the induction of sepsis, the loss of functional GPVI resulted in a significant reduction in the percentage of both classical and non‐classical monocytes associated with platelets (Figure 6d). Similar to the broad monocyte population, sepsis seemed to induce a dramatic increase in platelet‐classical and non‐classical monocyte aggregates in circulation in WT samples while GPVI^−/−^ appear unchanged indicating the relevance of GPVI in platelet‐monocyte aggregation formation under septic conditions.

### 3.7 GPVI regulation of monocyte subgroup activation and secretion during sepsis

**FIGURE 7:**
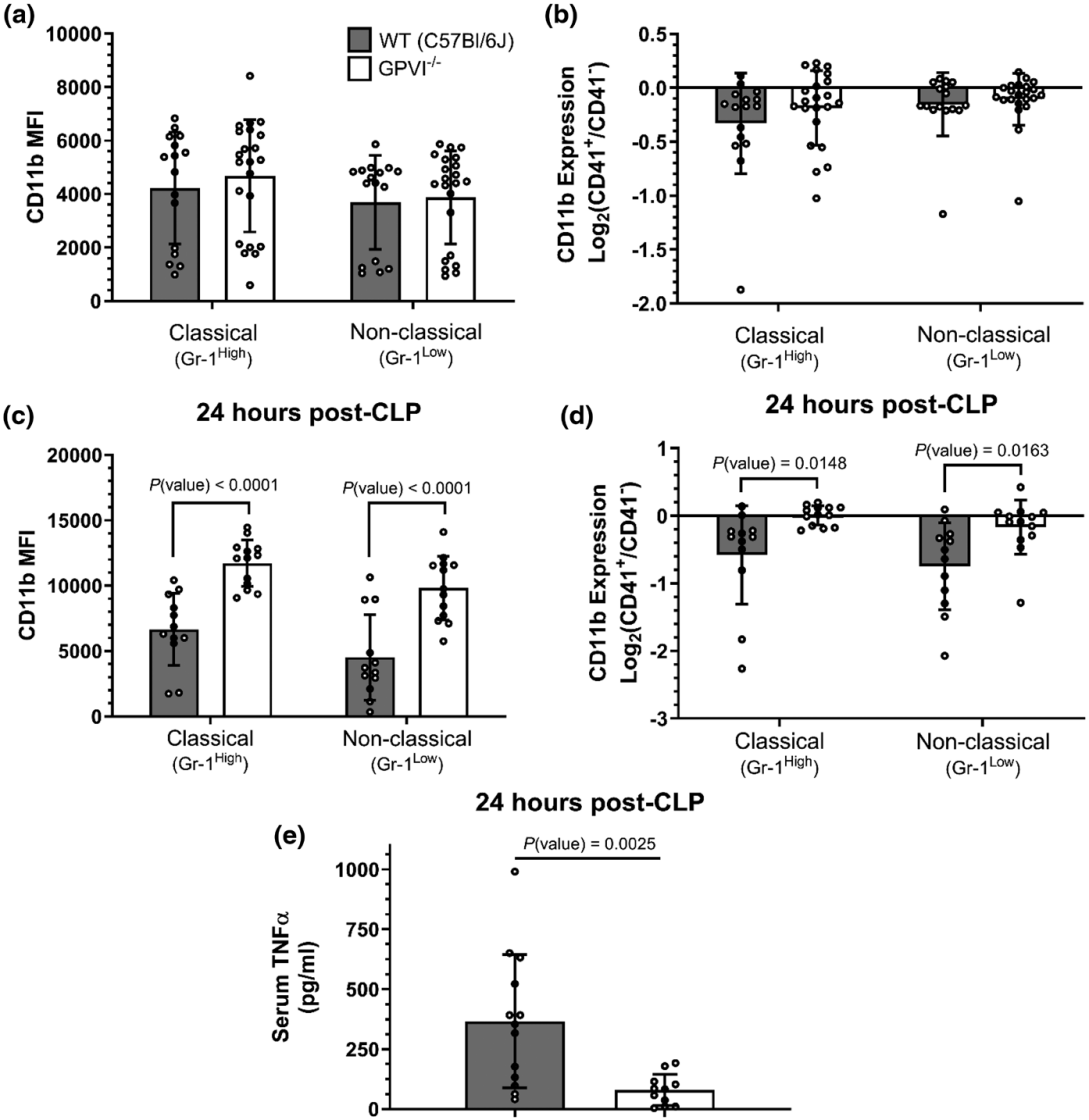
Monocyte sub‐type activation characteristics under normal and septic conditions. (a) CD11b surface expression profiles of classical and non‐classical monocytes prior to CLP. (b) The ratio of CD11b expression for CD41^+^ relative to CD41^−^ classical and non‐classical monocytes under non‐septic conditions. (c) CD11b expression for classical and non‐classical monocytes 24 h after CLP. (d) CD11b expression for classical and non‐classical monocytes 24 h post‐CLP expressed as a ratio of CD41^+^/CD41^−^ events. (e) Serum TNFα concentrations samples 24 h after sepsis initiation. CD11b characterization; Baseline conditions: *N* = 18 (WT); 25 (GPVI). Septic conditions: *N* = 14 (WT); 13 (GPVI^−/−^). TNFα quantification; *N* = 13 (WT); 11 (GPVI^−/−^).

The observation that GPVI governed significant changes in the composition of monocyte subgroups during sepsis directed us to next address any GPVI mediated impact on the activation status of these subtypes. Under baseline conditions, GPVI had no impact on the activation state of either classical or non‐classical monocytes (Figure 7a). Likewise, the shift in activation status facilitated by platelet adhesion to both sub‐classes trended downward relative to unbound monocytes with no difference noted between WT and GPVI^−/−^ (Figure 7b). However, following the induction of sepsis, GPVI^−/−^ classical and non‐classical monocytes exhibited marked increases in activation relative to WT (Figure 7c). Moreover, the result of platelet adhesion to both classical and non‐classical monocytes resulted in significantly attenuated activation relative to corresponding monocytes not associated with platelets in the presence of functional GPVI (Figure 7d). Of note, GPVI deletion negated any modulatory effect on monocyte activation as CD11b expression was mostly unchanged when comparing platelet‐bound and platelet free monocytes of both sub‐categories. We also measured serum TNFα at this stage of sepsis (24 h post‐CLP) to: (1) serve as a snapshot assessment of inflammatory status during sepsis due to its pleiotropic inflammatory effects; and (2) to serve as an assessment of monocyte pathophysiological function (Gharamti et al., 2022). Surprisingly, WT serum samples contained the highest concentration of TNFα indicating that while GPVI deletion elicits higher levels of non‐classical monocyte activation, that does not directly translate to enhanced secretory function (Figure 7e).

## 4 DISCUSSION

Sepsis is the resultant state of systemic inflammation caused by microbial penetrance of the circulatory system (Wiersinga et al., 2014). While inflammation is meant to be a defensive maneuver elicited primarily by leukocytes, the stratagems employed by immune cells are capable of damaging host tissues, which contributes to sepsis morbidity and mortality (Skirecki et al., 2012). Indeed, host immunity is a complex physiological process with a myriad of cellular and molecular participants, and so the brunt of research has addressed sepsis pathology by examining the core members of inflammation, with less emphasis on peripheral players such as platelets. Moreover, sepsis also elicits a disruption of hemostasis, causing platelet activation/consumption resulting in disseminated intravascular coagulation (DIC) and thrombocytopenia (Woth et al., 2011). And so, the primary focus of platelets in the context of sepsis has been through its hemostatic functionality and not its contribution to inflammation directly (Iba et al., 2023; Setarehaseman et al., 2025). Thus, the limited scope by which the platelet/sepsis axis is investigated represents a major shortcoming in the potential to curtail sepsis mortality.

Our data focusing on the platelet‐leukocyte interface during CLP‐induced systemic inflammation highlights previously unknown facets of the platelet‐specific receptor GPVI and its relevance to sepsis. Previously, GPVI was reported to elicit pro‐inflammatory microparticle formation, but this was merely an indirect affiliation with inflammation. To facilitate our investigation of a more direct involvement of GPVI in the context of inflammation, we began with a simple observation of GPVI's contribution to sepsis outcome broadly. Since GPVI deletion significantly impaired sepsis survival, we had to explore the potential that this outcome was the result of concomitant hemostatic impairment. Accordingly, platelet levels fluctuate in sepsis due to their removal from circulation via consumptive coagulopathy (van Vught et al., 2021). Paradoxically, this also heightens bleeding risk as mass activation clears platelets and impairs the maintenance of normal hemostasis, leading to vascular leakage. To address this, Evans Blue dye was used as a quantifiable proxy for blood volume, due to its association with circulating albumin, with elevated dye content in organs indicating extravascular leakage. When evidence of vascular leakage or alterations in platelet or red blood cell (RBC) counts resulting from GPVI deletion were not observed, we proceeded to focus on outlining potential variations in immunological characteristics attributed to GPVI as the primary driver of the heightened susceptibility to sepsis.

The formation of PLAs is a known avenue by which platelets directly govern leukocyte function and so we focused specifically on the association of platelets to neutrophils and monocytes as (1) these are the most well‐studied PLAs and (2) platelets demonstrate a preferential associate for these leukocytes relative to others (Rossaint et al., 2018). Baseline characterization of neutrophils and monocytes entangled with platelets revealed little deviations stemming from GPVI deletion. Likewise, documenting surface expression of Mac‐1 (CD11b), which is rapidly increased following stimulation, revealed no changes in leukocytes’ activation resulting from GPVI deletion (Meisel et al., 1998). Platelets, on the other hand, exhibited significantly lower levels of activation when neutrophil and monocyte aggregation was accompanied by GPVI deletion. As the crux of our investigation hinged upon the role of GPVI in inflammation, we re‐evaluated the same criteria under septic conditions. During sepsis GPVI deletion significantly diminished aggregate formation with neutrophils and monocytes, thereby alluding to a secondary role as an adhesion receptor. Moreover, while certain platelet and neutrophil characteristics were modified by GPVI deletion, our major finding was the sizeable influence on the monocyte phenotype. Indeed, GPVI loss reduced levels of platelet aggregate formation within the total monocyte population and its various subtypes. Interestingly, this deviation in aggregate formation was not due to a drop in levels for the GPVI^−/−^ relative to static conditions, but rather a GPVI‐driven increase in WT samples prompted by the onset of systemic inflammation.

This increased GPVI‐mediated platelet coverage of monocytes also served to direct monocyte polarization by enhancing the percentage of classical monocytes while diminishing the non‐classical pool. Furthermore GPVI+ platelets attenuated sepsis‐driven activation in the monocyte population as platelet binding drove down activation status relative to unassociated cells for both classical and non‐classical monocytes, while the binding of GPVI^−/−^ platelets had no discernible effect. Surprisingly, despite driving down monocyte activation and winnowing the non‐classical subtype, serum TNFα was substantially elevated in WT samples. Circulating TNFα is primarily released by non‐classical monocytes and serves to discern the functional consequence of GPVI modulation in this monocyte subtype (Mukherjee et al., 2015; Zelova & Hosek, 2013). And so, while GPVI can diminish the number of non‐classical monocytes and their broad activation profile, this does not translate to a hampering of all effector functions (i.e. cytokine secretion). Indeed, these results illustrate the nuanced nature by which effectors of inflammation and specific functions are delicately calibrated to mount an adequate immune response to pathogens.

For platelet GPVI to exert this level of influence over the leukocyte phenotype, there must be a binding partner present on immune cells. Indeed, Basigin (EMMPRIN, CD147) is a known counter‐receptor of GPVI expressed by leukocytes, but the nature of the relationship is obscure (Jouneau et al., 2011). Though our findings are by no means definitive, it is less likely that GPVI is facilitating these changes in leukocytes through some as of yet discovered binding partner in lieu of Basigin. Thus, we believe our findings provide early insight into the functional significance of the GPVI‐Basigin interface. Basigin signaling plays a role in TNFα secretion, and the attenuation of GPVI^−/−^ serum TNFα would indicate the necessity of this platelet receptor in facilitating that required signaling mechanism (Schmidt et al., 2008). While this is by no means a complete exploration of the pathophysiological function of the GPVI‐Basigin axis, it does identify future directions to determine the significance of this binding interaction.

In sum, mounting an inflammatory response to eliminate a microbial invasion requires tight regulation to prevent the cure from becoming more harmful than the disease. Our results suggest platelets are a part of this regulatory mechanism, with monocyte coverage increasing under septic conditions to serve as governors of inflammation. Non‐classical monocytes serve as “force‐multipliers” providing auxiliary, secretory support, allowing for a minimal population relative to the classical monocyte infantry, which functions as the phagocytic subgroup that infiltrates tissues and directly engages microbe invaders. Here, platelets utilize GPVI to increase the classical/non‐classical monocyte ratio, illustrating this delicate governance of sub‐population selective expansion. Additional restraints are also necessary to ensure that elimination of microbes does not result in collateral damage that places the host in dire straits. Again, platelets demonstrate this further regulatory functionality by limiting the activation status of classical/non‐classical monocyte via the binding of GPVI+ platelets. Despite this, the increase in serum TNFα represents a vexing characteristic in the face of this explanation. It would seem the nature of monocyte activation is much more nuanced than a binary “yes” or “no” state, with GPVI possibly diminishing certain activation characteristics and amplifying others. With TNFα exerting many downstream effects on a host of immune participants, maintaining a particular serum threshold is perhaps necessary in the broad context of an inflammatory response. So, it may be erroneous to attribute this snapshot observation of serum TNFα to our crude analysis of monocyte activation given the numerous interwoven inflammatory events that occur following sepsis onset and their dynamic fluctuations as time progresses.

## 5 LIMITATIONS

Given the dynamic and ever‐changing sequelae of events that lead to severe sepsis, our studies are not without limitations. Firstly, our documentation of platelet–neutrophil/monocyte aggregate parameters was cross‐sectional at 24 h post CLP. Given the dynamic, temporal sequence of events that drive sepsis pathophysiology, it is pertinent that future studies encompass multiple time points following the induction of sepsis to provide details as to whether the observed characteristics are persistent or fluctuate temporally. Next, we conducted our experiments utilizing only males, which limits the scope of applicability of these findings. As discussed beforehand, we focused exclusively on males as they are the cohort routinely documented to have a significantly enhanced disease burden in sepsis relative to females (Shields et al., 2023). When designing the initial experiments, we chose to study males because they exhibit greater susceptibility to sepsis and therefore were expected to show a clearer phenotype following GPVI deletion. We reasoned that any effects of GPVI on systemic inflammation would be more apparent in a model (males) with more severe sepsis. However, it remains unclear whether the improved sepsis outcomes observed in females are driven by differences in inflammatory response and whether GPVI plays a similar or distinct role in females compared with males. Female responses may indeed differ from those of males, as reflected by differences in sepsis survival rates, but further investigations are needed to determine whether inflammatory pathways and GPVI contribute to these observed sex differences. It is also worth noting that we limited our characterization of platelet associations to neutrophils and monocytes. Though the platelet's preferential binding to neutrophils and monocytes is widely noted, other leukocyte sub‐categories are also capable of aggregating with platelets (Finsterbusch et al., 2018). And so, it is necessary to broaden our investigation to encompass characterization of several other PLA variants to provide a more comprehensive assessment of the GPVI contribution to the sepsis‐induced inflammatory response. In addition, while we found no difference in blood cell counts or extravascular tissue leakage, we did not substantially explore other avenues of hemodynamic perturbations. Indeed, while our primary objective was to discern the potential contribution of GPVI to the inflammatory response, there is still the potential that alterations to hemostasis/thrombosis due to GPVI deletion could explain the difference in septic survival. As such, measuring prothrombin time, serum D‐dimer levels, or platelet aggregation in septic samples is a necessary future direction to supplement our findings. Despite these limitations, the results of this study provide a critical foundation into the continued exploration of the significance of platelets and platelet‐specific receptors as key players in inflammation.

## 6 CONCLUSION

The continuous overlap of inflammation and platelet functionality signifies the consideration of platelets as an extension of the immune system. Thus, reevaluating platelet traits through the framework of inflammation opens up new potential for better understanding vexing pathways that have resulted in a stagnation to curtail certain inflammatory disorders such as sepsis. Certainly, the broad array of receptors housed on the platelet membrane provides several opportunities to investigate platelet relevance in a multitude of inflammatory settings. By deducing which mechanism under which setting is influenced by these receptors, new studies will likely generate more appropriately tailored therapies for specific pathology. By accepting the platelet as a relevant component of the immune system, we can move one step further towards unraveling the complex pathophysiology of inflammation and provide much needed improvement in the treatment of not only sepsis but all inflammatory disorders.

## AUTHOR CONTRIBUTIONS

**Adam Corken:** Conceptualization; data curation; formal analysis; investigation; methodology. **Jerry Ware:** Conceptualization; formal analysis; funding acquisition; project administration; supervision. **Keshari M. Thakali:** Formal analysis; project administration; supervision; visualization.

## FUNDING INFORMATION

This research was supported by funding from the NIH – Leukocyte‐platelet modulation of the systemic inflammatory response (5R21AI133160‐02).

## CONFLICT OF INTEREST STATEMENT

The authors declare that they have no known competing financial interests or personal relationships that could have appeared to influence the work reported in this paper.

## ETHICS STATEMENT

The animal study protocol was approved by the University of Arkansas for Medical Sciences Institutional Animal Care and Use Committee (IPROTO202400000124, approved 1/14/2020).

## Supporting information


**Figure S1:** Flow cytometry gating strategy to visualize leukocyte populations in whole blood. (a) A “cell” gate was established to eliminate smaller particles and instrument noise based on size (FSC) and granularity (SSC) characteristics. (b) Within the “cell” population, Gr‐1 and CD115 staining was used in the determination of the neutrophil/PMN (Gr‐1^+^/CD115^−^) and monocyte (CD115^+^) populations. Platelet adherence was determined for (c) neutrophil and (d) monocyte events that expressed CD41^+^ (platelet) staining. (e) The monocyte population was further subcategorized based on GR‐1 expression to establish the “classical” (Gr‐1^High^) and “non‐classical” (Gr‐1^Low^) subsets.
**Figure S2:** Flow cytometry gating analysis to determine cellular associations of the platelet population. (a) The platelet population was determined by visualizing a non‐excluded data sample and then gating onto CD41^+^ population. As platelets are smaller than the typical cell events, prior exclusion of events by gating onto a traditional “cell” population was avoided. Furthermore, as we wanted to account for platelets adherent to neutrophils and monocytes, the gate was drawn across the entire spectrum of the FSC axis rather than limiting the gating to platelet‐sized events which would eliminate all other blood cells from analysis. (b) Using Gr‐1 and CD115 fluorescence within the platelet‐positive population would then allow for the determination of PMN/neutrophil and monocyte positive events which were interpreted as platelet–neutrophil/monocyte aggregates. Additionally the absence of Gr‐1 and CD115 staining was utilized to gate onto an unbound/free platelet population.
**Figure S3:** Representative dot plots of CD41^+^ neutrophils and monocyte populations. The (a) neutrophil and (b) monocyte population was identified for both WT and GPVI^−/−^ samples and the percentage of platelet positive events (CD41^+^) was determined by documenting the events that crossed a predetermined fluorescent threshold (dotted line).
**Figure S4:** Representative determination of activation levels for leukocyte and platelet populations. (a) Within a specified leukocyte subset, CD11b fluorescent values were obtained and compared between WT and GPVI^−/−^ samples to illustrate differing levels of activation resulting from GPVI deletion. (b) Platelet activation resulting from aggregate formation with neutrophils/monocyte corresponding to the presence or absence of GPVI was evaluated by observing P‐selectin fluorescence.

## Data Availability

The dataset outlined in this manuscript is available from the corresponding author upon request.
