# Supplementary material for: Platelet glycoprotein VI modulates leukocyte characteristics in sepsis‐induced systemic inflammatory response in male mice
Source: Physiol Rep. 2026 Jun 16;14(12):e70971. doi: 10.14814/phy2.70971 (PMC13273020; doi:10.14814/phy2.70971)

Supplementary Material

Platelet Glycoprotein VI Modulates Leukocyte Characteristics in Sepsis Induced Systemic Inflammatory Response in Male Mice

**Authors:** Adam Corken^1,2^**,** Jerry Ware^3^ and Keshari M. Thakali^1,2^

^1^Department of Pediatrics, University of Arkansas for Medical Sciences, Little Rock, AR, USA;

^2^Arkansas Children’s Research Institute, Little Rock, AR, USA

^3^Department of Physiology and Biophysics, University of Arkansas for Medical Sciences, Little Rock, AR


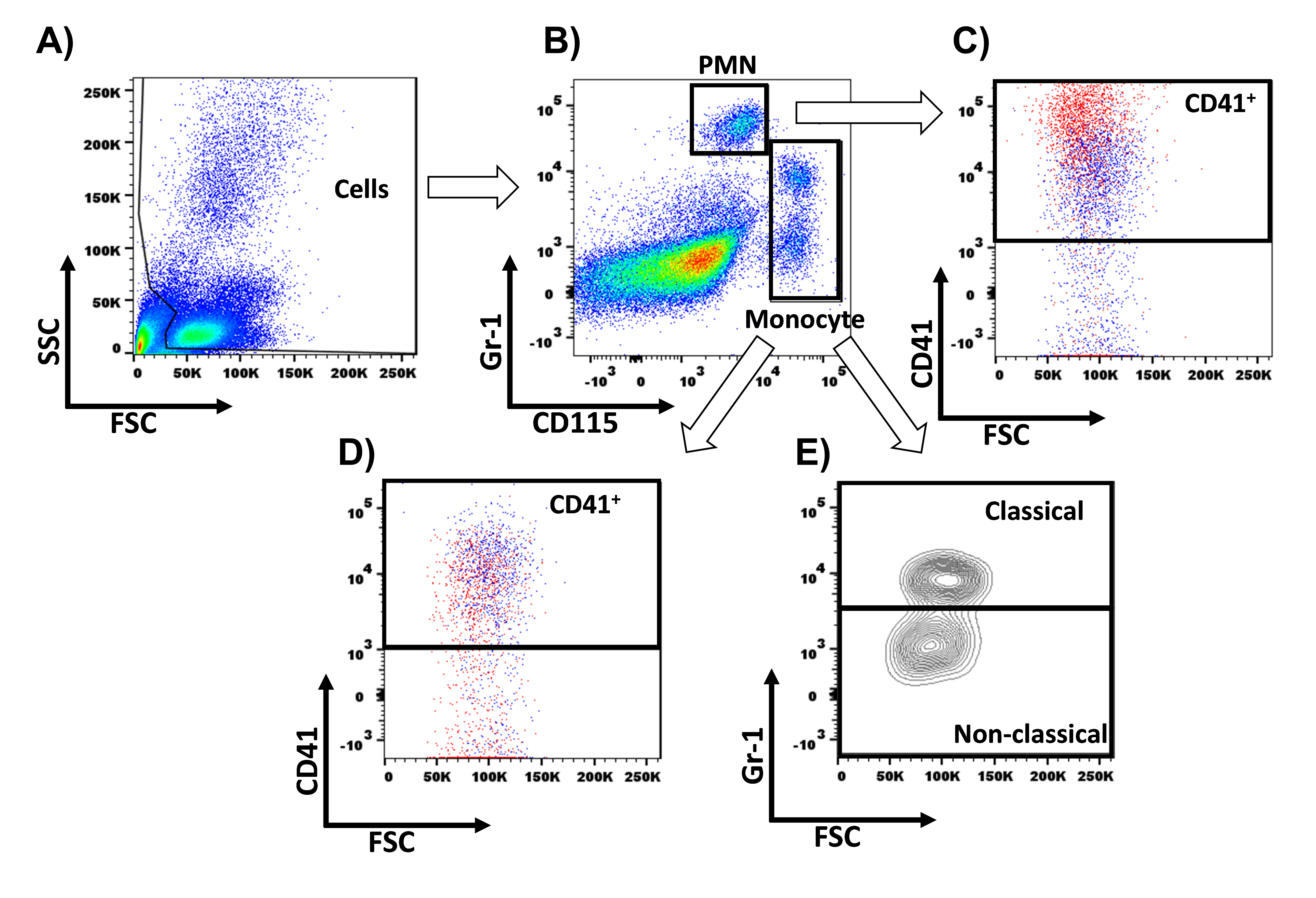


**Figure 1: Flow cytometry gating strategy to visualize leukocyte populations in whole blood. A)** A “cell” gate was established to eliminate smaller particles and instrument noise based on size (FSC) and granularity (SSC) characteristics. **B)** Within the “cell” population, Gr-1 and CD115 staining was used in the determination of the neutrophil/PMN (Gr-1^+^/CD115^-^) and monocyte (CD115^+^) populations. Platelet adherence was determined for **C)** neutrophil and **D)** monocyte events that expressed CD41^+^ (platelet) staining. **E)** The monocyte population was further subcategorized based on GR-1 expression to establish the “classical” (Gr-1^High^) and “non-classical” (Gr-1^Low^) subsets.

**Figure 2: Flow cytometry gating analysis to determine cellular associations of the platelet population. A)** The platelet population was determined by visualizing a non-excluded data sample and then gating onto CD41^+^ population. As platelets are smaller than the typical cell events, prior exclusion of events by gating onto a traditional “cell” population was avoided. Furthermore, as we wanted to account for platelets adherent to neutrophils and monocytes, the gate was drawn across the entire spectrum of the FSC axis rather than limiting the gating to platelet-sized events which would eliminate all other blood cells from analysis. **B)** Using Gr-1 and CD115 fluorescence within the platelet-positive population would then allow for the determination of PMN/neutrophil and monocyte positive events which were interpreted as platelet-neutrophil/monocyte aggregates. Additionally the absence of Gr-1 and CD115 staining was utilized to gate onto an unbound/free platelet population.


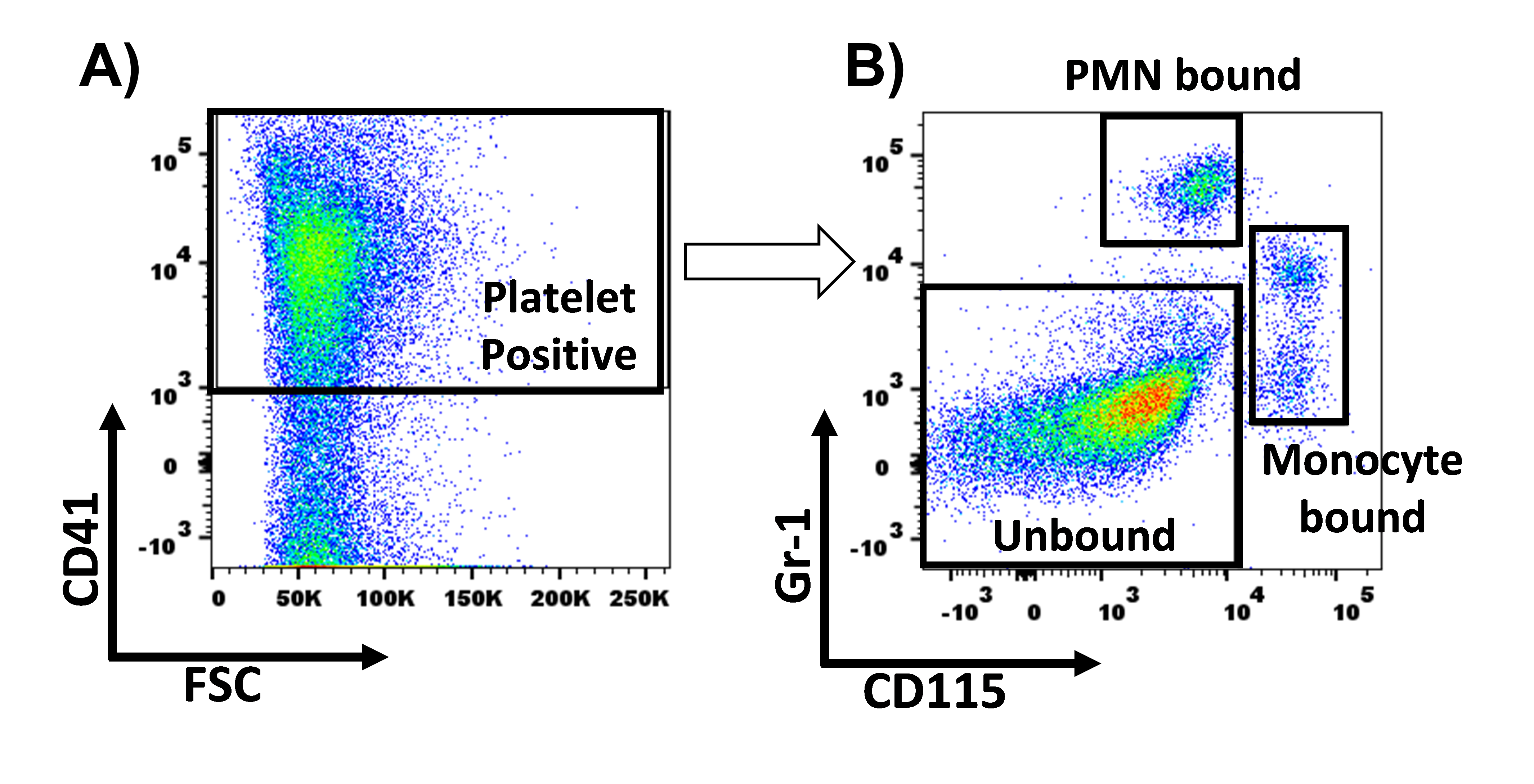

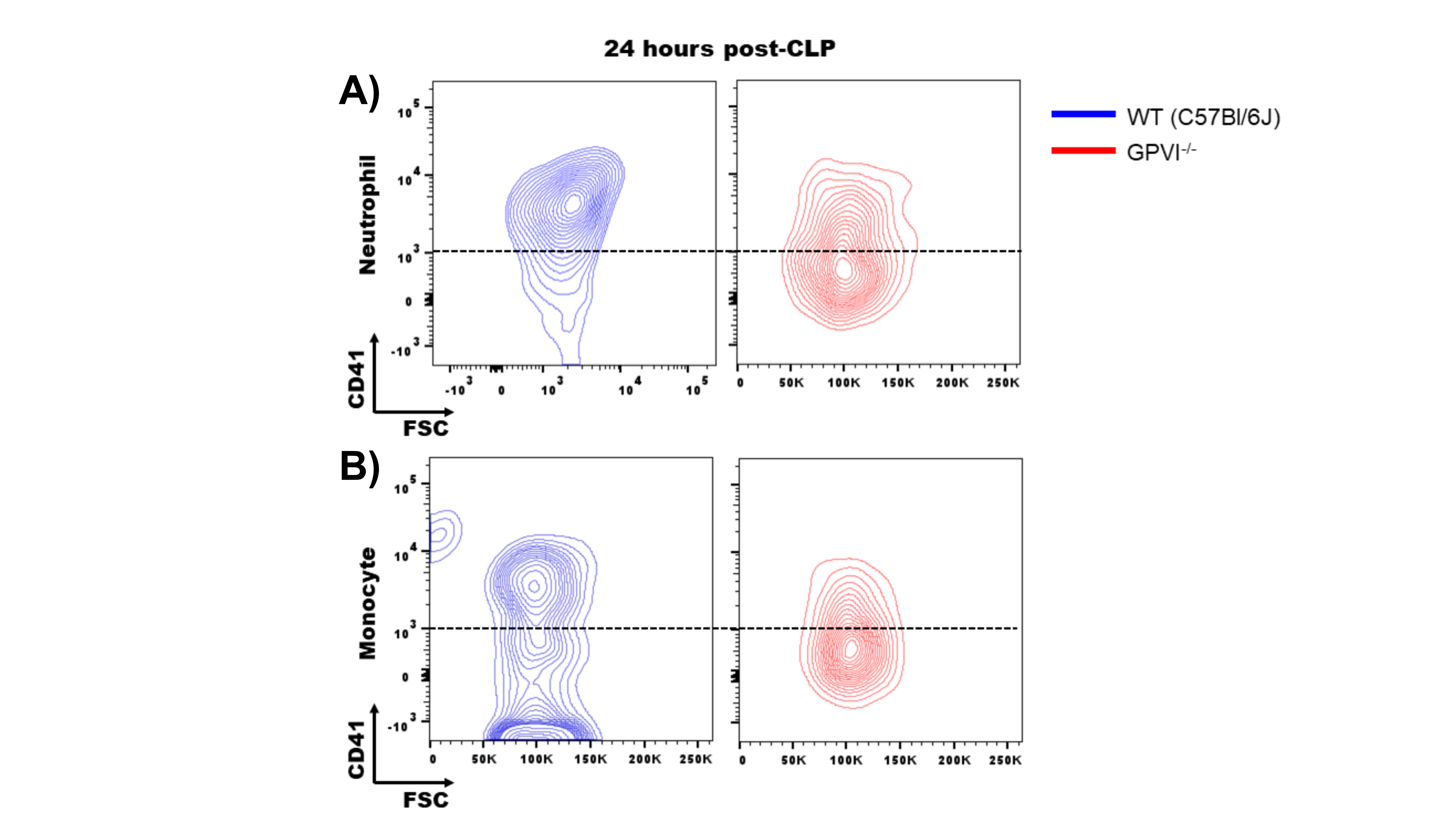


**Figure 3: Representative dot plots of CD41^+^ neutrophils and monocyte populations.** The **A)** neutrophil and **B)** monocyte population was identified for both WT and GPVI^-/-^ samples and the percentage of platelet positive events (CD41^+^) was determined by documenting the events that crossed a predetermined fluorescent threshold (dotted line).

**Figure 4: Representative determination of activation levels for leukocyte and platelet populations.** **A)** Within a specified leukocyte subset, CD11b fluorescent values were obtained and compared between WT and GPVI^-/-^ samples to illustrate differing levels of activation resulting from GPVI deletion. **B)** Platelet activation resulting from aggregate formation with neutrophils/monocyte corresponding to the presence or absence of GPVI was evaluated by observing P-selectin fluorescence.


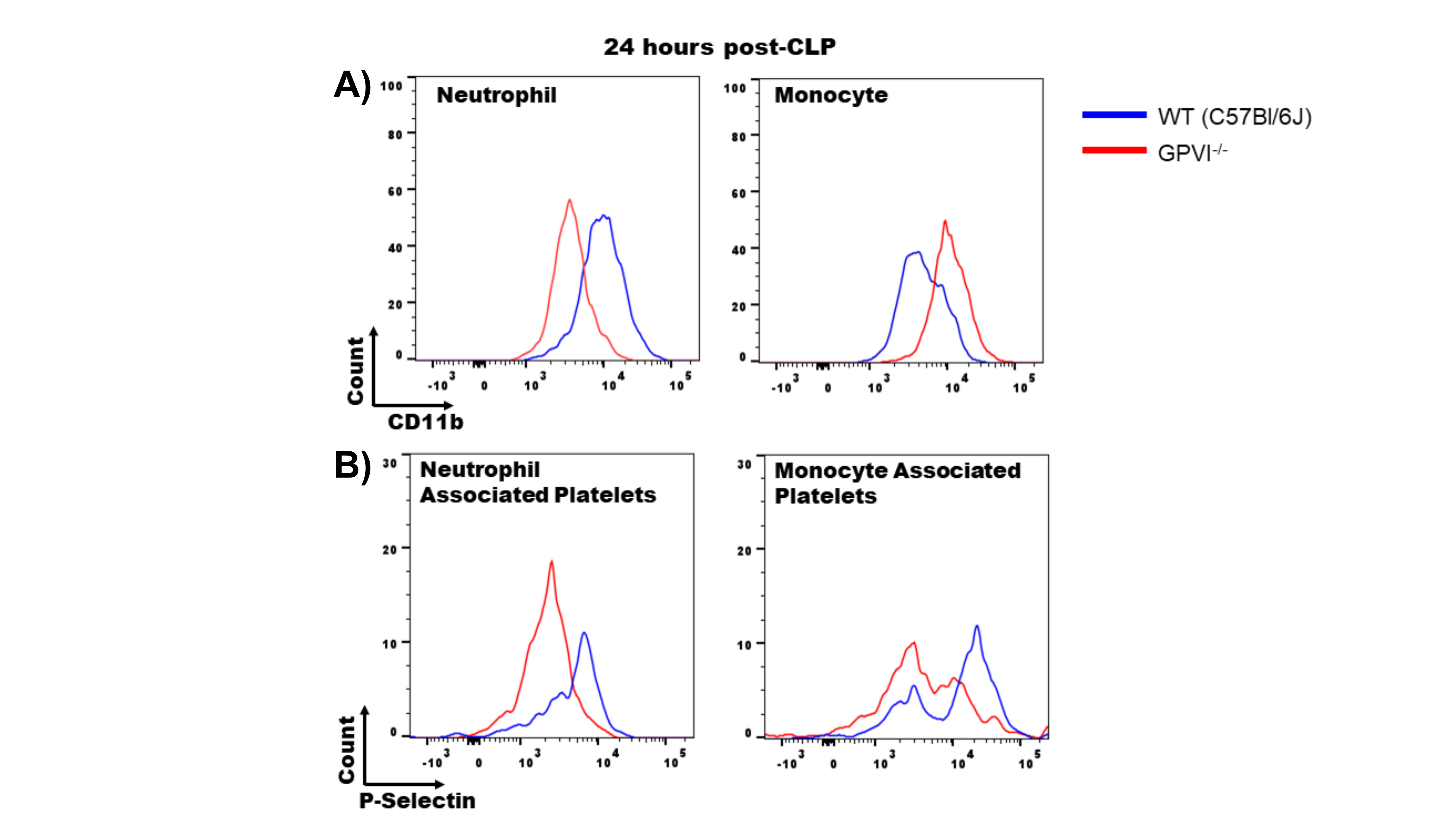

Supplement: Supplementary file 1 — Figure S1: Flow cytometry gating strategy to visualize leukocyte populations in whole blood. (a) A “cell” gate was established to eliminate smaller particles and instrument noise based on size (FSC) and granularity (SSC) characteristics. (b) Within the “cell” population, Gr‐1 and CD115 staining was used in the determination of the neutrophil/PMN (Gr‐1+/CD115−) and monocyte (CD115+) populations. Platelet adherence was determined for (c) neutrophil and (d) monocyte events that expressed CD41+ (platelet) staining. (e) The monocyte population was further subcategorized based on GR‐1 expression to establish the “classical” (Gr‐1High) and “non‐classical” (Gr‐1Low) subsets. Figure S2: Flow cytometry gating analysis to determine cellular associations of the platelet population. (a) The platelet population was determined by visualizing a non‐excluded data sample and then gating onto CD41+ population. As platelets are smaller than the typical cell events, prior exclusion of events by gating onto a traditional “cell” population was avoided. Furthermore, as we wanted to account for platelets adherent to neutrophils and monocytes, the gate was drawn across the entire spectrum of the FSC axis rather than limiting the gating to platelet‐sized events which would eliminate all other blood cells from analysis. (b) Using Gr‐1 and CD115 fluorescence within the platelet‐positive population would then allow for the determination of PMN/neutrophil and monocyte positive events which were interpreted as platelet–neutrophil/monocyte aggregates. Additionally the absence of Gr‐1 and CD115 staining was utilized to gate onto an unbound/free platelet population. Figure S3: Representative dot plots of CD41+ neutrophils and monocyte populations. The (a) neutrophil and (b) monocyte population was identified for both WT and GPVI−/− samples and the percentage of platelet positive events (CD41+) was determined by documenting the events that crossed a predetermined fluorescent threshold (dotted line). F [file PHY2-14-e70971-s001.docx]
